# CiLiQuant: Quantification of RNA Junction Reads Based on Their Circular or Linear Transcript Origin

**DOI:** 10.3389/fbinf.2022.834034

**Published:** 2022-02-22

**Authors:** Annelien Morlion, Eva Hulstaert, Marieke Vromman, Jasper Anckaert, Celine Everaert, Jo Vandesompele, Pieter Mestdagh

**Affiliations:** ^1^ Department of Biomolecular Medicine, Ghent University, Ghent, Belgium; ^2^ OncoRNALab, Cancer Research Institute Ghent (CRIG), Ghent, Belgium; ^3^ Translational Oncogenomics and Bioinformatics Lab, Cancer Research Institute Ghent (CRIG), Ghent, Belgium

**Keywords:** RNA, circular, linear, splicing, bioinformatics

## Abstract

Distinguishing circular RNA reads from reads derived from the linear host transcript is a challenging task because of sequence overlap. We developed a computational approach, CiLiQuant, that determines the relative circular and linear abundance of transcripts and gene loci using back-splice and unambiguous forward-splice junction reads generated by existing mapping and circular RNA discovery tools.

## Introduction

Circular RNAs (circRNAs) are a novel class of non-coding RNAs found in eukaryotic transcriptomes that result from a process called back-splicing during RNA maturation. In recent years, circRNAs are attracting considerable research attention and evidence of their involvement in normal development and disease has been reported (Maass et al., 2017; Gaffo et al., 2019; Vo et al., 2019). Due to their stable, circular conformation, tissue-specific expression patterns and abundance in biofluids, circRNAs are emerging as potential biomarker candidates in minimally-invasive liquid biopsies (Salzman et al., 2013; Zhang et al., 2018; Su et al., 2019; Hulstaert et al., 2020). As circRNAs share most of their sequence with their linear counterparts, it is impossible to uniquely assign massively parallel RNA sequencing reads to linear or circular transcripts if the read does not include the back-splice sequence itself. This hampers calculations of the relative contribution of circRNAs to aggregated gene counts and can obscure differential expression analyses. To this end, we developed a computational pipeline that builds on the output of common mapping strategies to determine the linear or ambiguous character of forward junctions. Based on this classification, we also propose two strategies to determine the circular fraction for a region of interest.

## Methods

### CiLiQuant Pipeline

**FIGURE 1:**
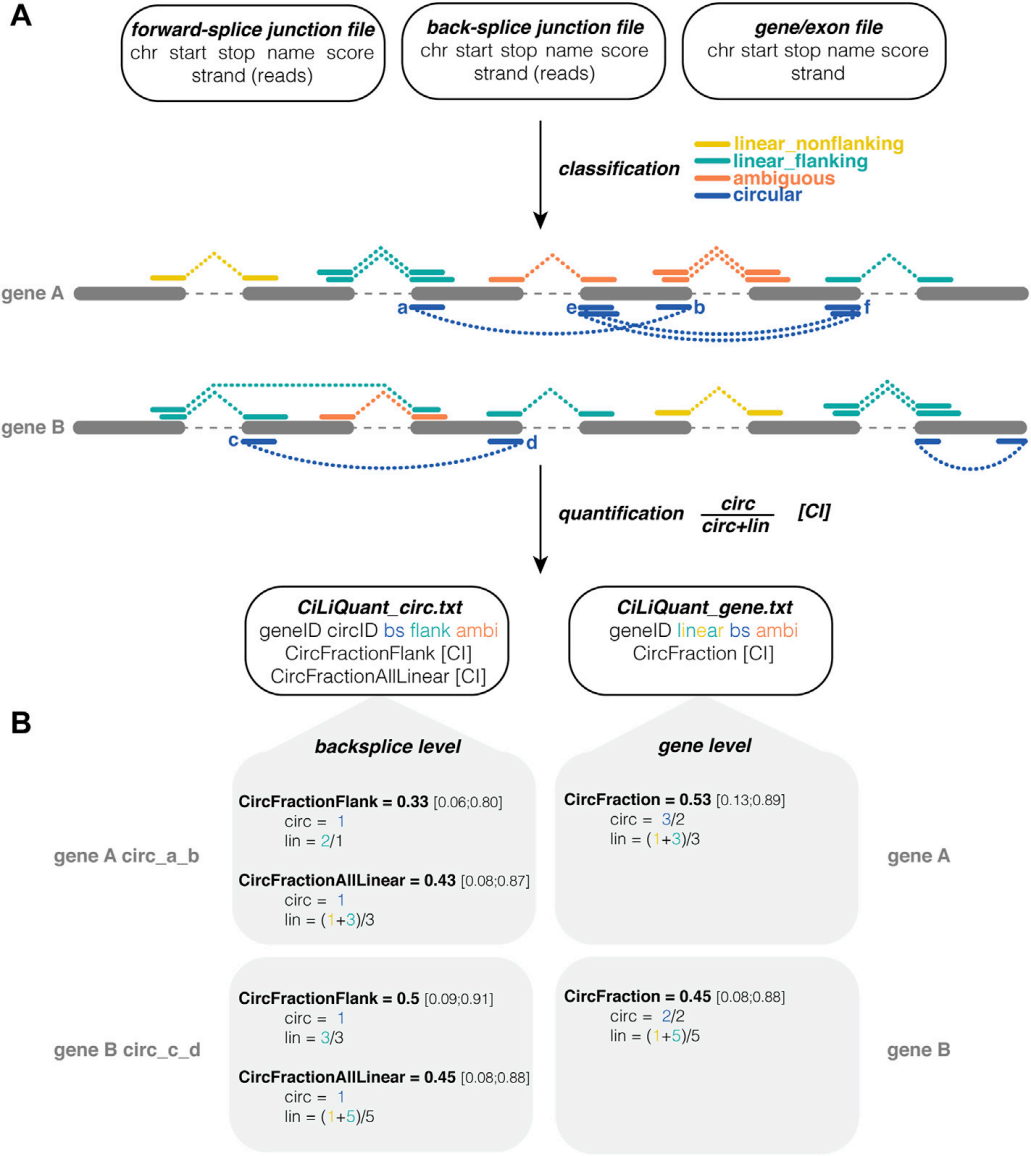
CiLiQuant classifies splice junctions based on their linear or circular origin and determines circRNA fractions. **(A)** Input, processing and output of the pipeline. Three input files required (number of junction reads may be in separate column or in score column), junctions are divided into four types based on overlap with detected circRNAs, quantification both at back-splice and gene level; **(B)** Examples of circRNA fraction calculations. CircFractionFlank only considers linear junctions directly next to the back-splice of interest (linear_flanking). CircFractionAllLinear and CircFraction consider all linear (linear_flanking and linear_nonflanking) junctions in the gene. In each calculation the sum of junction reads is corrected for the number of distinct junctions. Note that the counts in this example are rather low resulting in large confidence intervals. Test case with real sequencing data on GitHub. ambi: ambiguous (circular or linear origin); bs: back-splice; chr: chromosome; CI: Agresti-Coull 95% confidence interval; linear_flanking: forward-splice that partially overlaps with a back-splice junction (starts outside and stops inside the back-splice interval or vice versa); linear_nonflanking: forward-splice that shows no overlap with a back-splice junction.

The pipeline requires three input files in tab-separated format. Any combination of existing mapping and circRNA discovery tools can be used to generate the first two files: one file for forward-splice junctions, e.g., from STAR (Dobin et al., 2013), and one for back-splice junctions e.g. from CIRCexplorer2 (Zhang et al., 2016). The only requirements are that these junction files were generated from the same sequencing data using the same reference genome and that the files contain information about the coordinates of the junctions and their respective read counts in separate columns. The third input file should contain start and stop coordinates of the genes (or exons) of interest. The pipeline can be initiated using a single command and only requires Python and the Pandas package (The Pandas Development Team, 2010). The pipeline has a strand specific as well as an unstranded mode. Users can also impose a minimal count threshold to ignore forward-splice and back-splice junctions with less supporting reads than this threshold. More details and example input files can be found on GitHub (https://github.com/OncoRNALab/CiLiQuant). For each gene, the pipeline classifies forward-splice junctions as having a linear or ambiguous origin based on their overlap with detected back-splice junctions (Figure 1A). A forward-splice junction that starts and stops in between any detected back-splice junction is considered ambiguous because both linear and circular RNA can contribute to the read counts of this junction. In case the forward-splice junctions do not (completely) fall within the start and stop of any detected back-splice junction, they are classified as linear.

Equation 1: Circular fraction

$$Circular\;fraction=\frac{circ}{circ+lin}with\;circ=\frac{\sum \;backsplice\;junction\;reads}{\#\;backsplice\;junctions}and\;\;lin\;=\frac{\sum \;linear\;only\;junction\;reads}{\#\;linear\;only\;junctions}$$ (1)

Using this information, our method determines the relative circular to linear RNA abundance at two levels, back-splice and gene level (Figure 1B). At the gene level, this circular fraction is calculated by comparing the average number of back-splice junction reads to the average number of linear only junction reads in the gene (Eq. 1). At the individual back-splice level, reads of a back-splice junction are compared to the average of linear only forward-splice junction reads that are directly flanking the back-splice. The circular fraction is calculated as in Eq. 1, but “lin” is limited to those particular flanking junctions. Of note, sometimes this flanking information is not available—either because there are no flanking reads or because those reads are classified as ambiguous as they could be derived from another circRNA. Therefore, an alternative calculation using the average of all linear only junction reads in the gene is provided as well. This alternative calculation compares reads of a back-splice junction to the average of all linear only forward-splice junction reads in the corresponding gene. For each circular fraction, an Agresti-Coull 95% confidence interval is calculated (Brown et al., 2001).

### Cell Line Data Collection

HLF (hepatocellular liver carcinoma) and NCI-H23 (non-small cell lung adenocarcinoma) cells were cultured at 37°C, 5% CO2 in DMEM, low glucose, GlutaMAX Supplement, pyruvate (21,885,025, ThermoFisher) and RPMI 1640 Medium, HEPES (52,400,041, ThermoFisher), respectively. 10% fetal bovine serum (FBS) (F7524, Sigma) and 1% Penicillin-Streptomycin (10,000 U/mL) (15,140,122, ThermoFisher) were added to both media. RNA was isolated from the cells using the miRNeasy Mini kit (217,004, Qiagen) according to the manufacturer’s instructions, including an on-column DNase treatment (79,254, Qiagen). After isolation, the RNA concentration was measured (NanoDrop) and the RNA quality was evaluated (Fragment Analyzer). For each cell line, the RNA was first pooled and then divided into two samples, each containing 1 µg RNA in 100 µL nuclease-free water. First, ribosomal RNA (rRNA) was removed with the NEBNext rRNA Depletion Kit (E6350X, New England Biolabs), following the manufacturer’s instructions. Next, Ribonuclease R (RNAse R) treatment (RNR07250 (250 U), Lucigen) was performed according to our previously described protocol (Vromman et al., 2021). In summary, one sample of each cell line was treated with RNase R and one sample of each cell line was treated as a buffer-control. This was followed by a clean-up step using Vivacon 500, 10,000 MWCO Hydrosart columns (VN01H02, Sartorius). Next, the NEBNext Ultra II Directional RNA Library Prep Kit for Illumina (E7760L, New England Biolabs) was used in combination with the NEBNext Multiplex Oligos for Illumina (E7600S, New England Biolabs) to index and prep the samples. The protocol was adjusted to obtain relatively long insert sizes (RNA fragmentation of 7.5 min; first-strand cDNA synthesis elongation step of 50 min instead of 15). The last bead clean-up step was performed twice to completely remove all indexes from the samples. Finally, the samples were pooled and sequenced on a NovaSeq 6000 instrument using a NovaSeq 6000 S1 Reagent Kit v1.5 (300 cycles) (20028317, Illumina), resulting in approximately 300M paired-end reads per sample. Raw FASTQs are available at SRA (PRJNA789110). Only reads passing quality control (base calling accuracy of ≥99% in at least 80% of the nucleotides in both mates of a pair) were included in this analysis. RNA sequencing data was further subsampled to 10, 25, and 50 M paired-end reads using Seqtk v1.3 (Li, 2012). Finally, CiLiQuant without filter was applied to the forward-splice and back-splice junction file generated by STAR (Dobin et al., 2013) and CircExplorer2 (Zhang et al., 2016) using UCSC GRCh38/hg38 as reference genome. In each sample, at least 98% of all reads could be mapped (92% and 76–81% of reads were uniquely mapped in the untreated and RNase R treated samples, respectively). CiLiQuant output tables for 50M paired-end reads per sample can be found in Supplementary Material.

## Results

**FIGURE 2:**
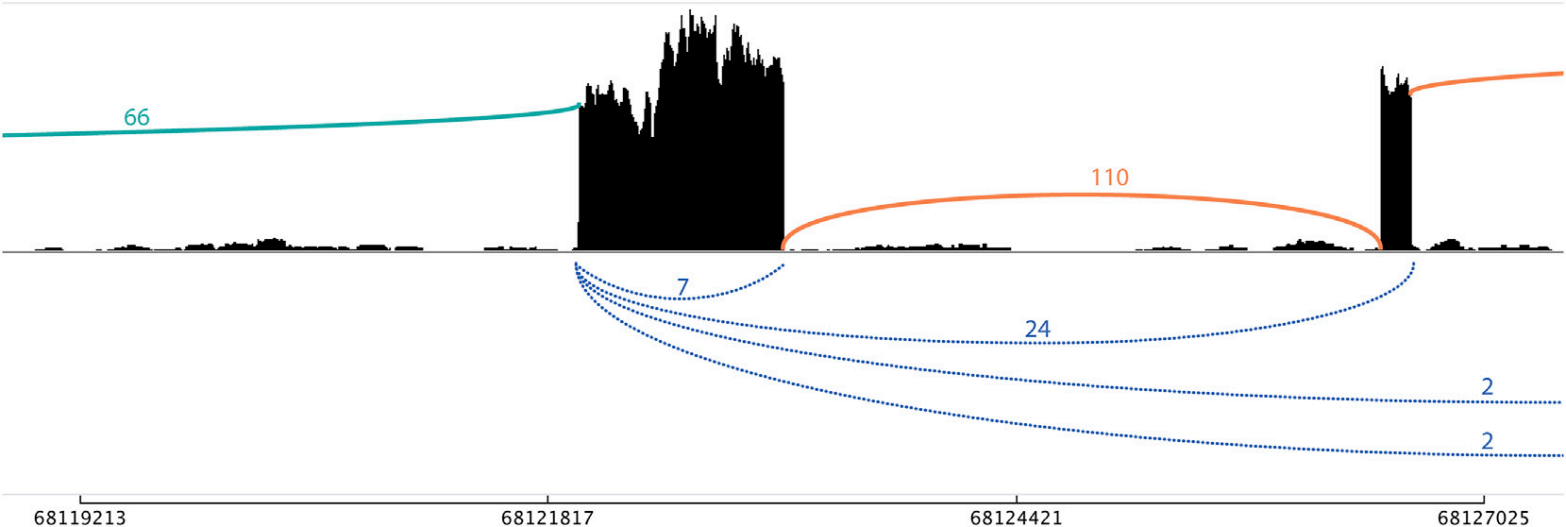
Sashimi plot with linear only and ambiguous flanking junctions of *NFATC3* in HLF cells. A single-exon circRNA (chr16_68121986_68123121_+) has seven supporting back-splice reads (blue). The 66 forward-splice junction reads on the left do not fall within other detected back-splice junctions and are therefore classified as linear only (green). The 110 forward-splice junction on the right are ambiguous (orange) as can be derived from both linear and circular transcripts [with 24, 2, and 2 back-splice reads supporting the back-splice junctions that span these forward-splice junctions, respectively (blue)].

**FIGURE 3:**
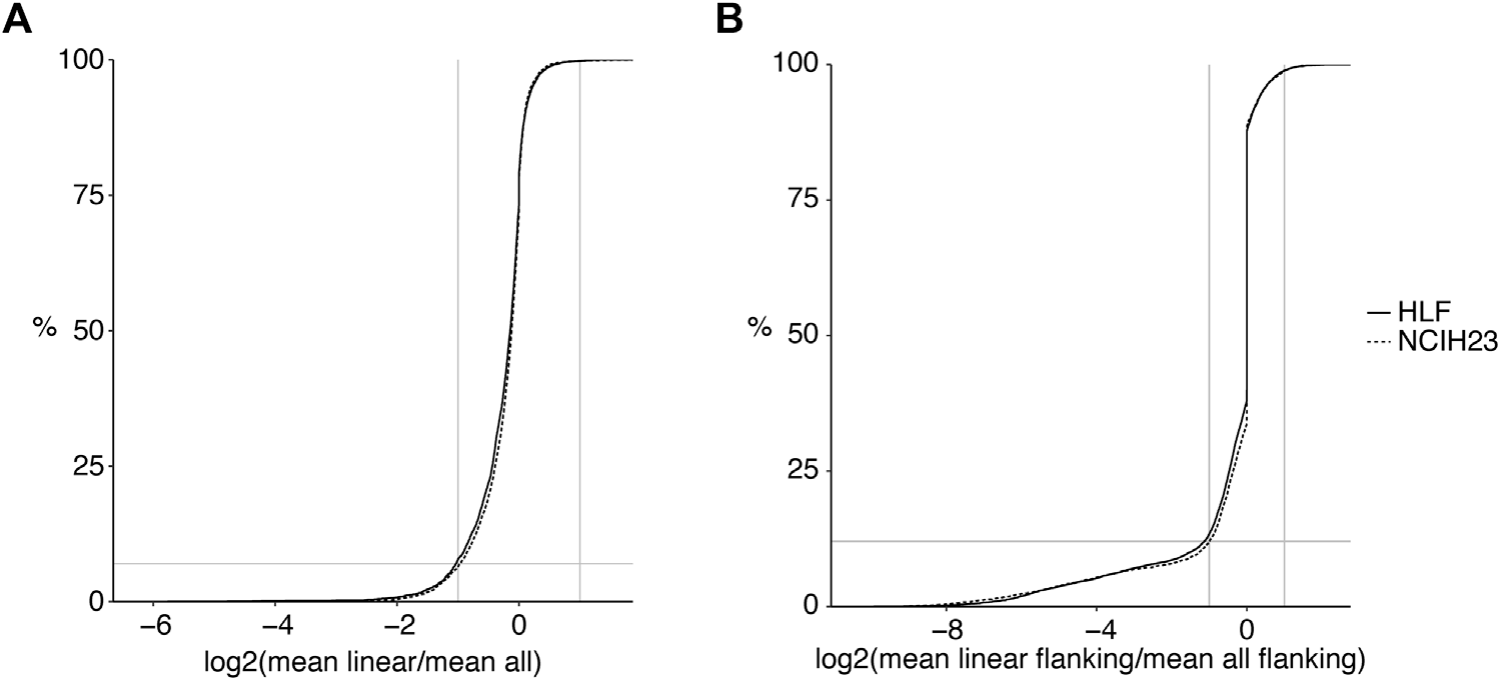
Linear transcript counts can be overestimated in genes with circular transcripts. **(A)** Log2 fold changes of linear only reads per junction (CiLiQuant) compared to all forward-splice reads per junction (STAR mapping) for genes that have at least one back-splice junction (3,889 and 3,897 genes in HLF and NCIH23, respectively). Horizontal grey line: 8%; **(B)** At individual back-splice level, log2 fold changes of flanking linear only reads per junction (CiLiQuant) compared to all flanking forward-splice reads per junction (STAR mapping). Only backsplice junctions that have linear only flanking junctions are shown (8,186 in HLF, 7,480 in NCIH23). Horizontal grey line: 12%. Vertical grey lines: absolute fold changes of 2.

CiLiQuant determines whether detected forward-splice junction reads have an ambiguous (linear or circular) origin by looking at overlap with detected back-splice junctions (Figure 1A). The classification is important for genes that have multi-exon or overlapping circular transcripts because their linear forward-splice junction count may be overestimated. Figure 2 illustrates this problem for junctions of *NFATC3* in HLF cells. In the total RNA-sequencing data from HLF and NCIH23 cell lines, 20% of genes that have at least one forward-splice junction read have at least one back-splice junction read as well (3,889 and 3,897 genes, respectively). For those genes with circular transcripts, we observed that the average of true linear counts per junction can be at least 2 times overestimated in 7–8% of the genes compared to the average of all forward-splice counts per junction obtained with STAR mapping (Figure 3A). The impact of the ambiguous reads is even more prominent at individual back-splice level. In HLF, 9,384 unique back-splice junctions with flanking forward-splice reads are detected. For 13% of those back-splice junctions, all flanking junctions are ambiguous which means that we cannot be sure whether the flanking reads are from linear or circular transcripts. CiLiQuant here provides the alternative of comparing back-splice reads to the average of linear only forward-splice reads per junction in the entire gene. For an additional 12% of back-splice junctions, the average linear only reads per flanking junction can be at least two times overestimated compared to the average of all forward splice reads per flanking junction (Figure 3B). Similar results were obtained in NCIH23: 8,556 unique back-splice junctions with flanking forward-splice reads detected, 13% of those back-splice junctions only have ambiguous flanking junctions and in 10% of back-splice junctions the linear only flanking reads per junction can be overestimated at least two times (Figure 3B).

CiLiQuant also calculates circular-to-linear fractions at both the gene and individual back-splice level (Figure 1B). The back-splice level calculation gives an idea of the frequency of back-splicing compared to forward-splicing in that specific region. Circular fractions at gene level can be used to determine to which extent (part of) the gene is expressed in a circular form. A non-zero circular fraction indicates that read counts of this gene are not merely coming from linear transcripts. This can be important for samples such as biofluids where the circular extracellular RNAs seem to be better preserved than their linear counterparts (Hulstaert et al., 2020).

**FIGURE 4:**
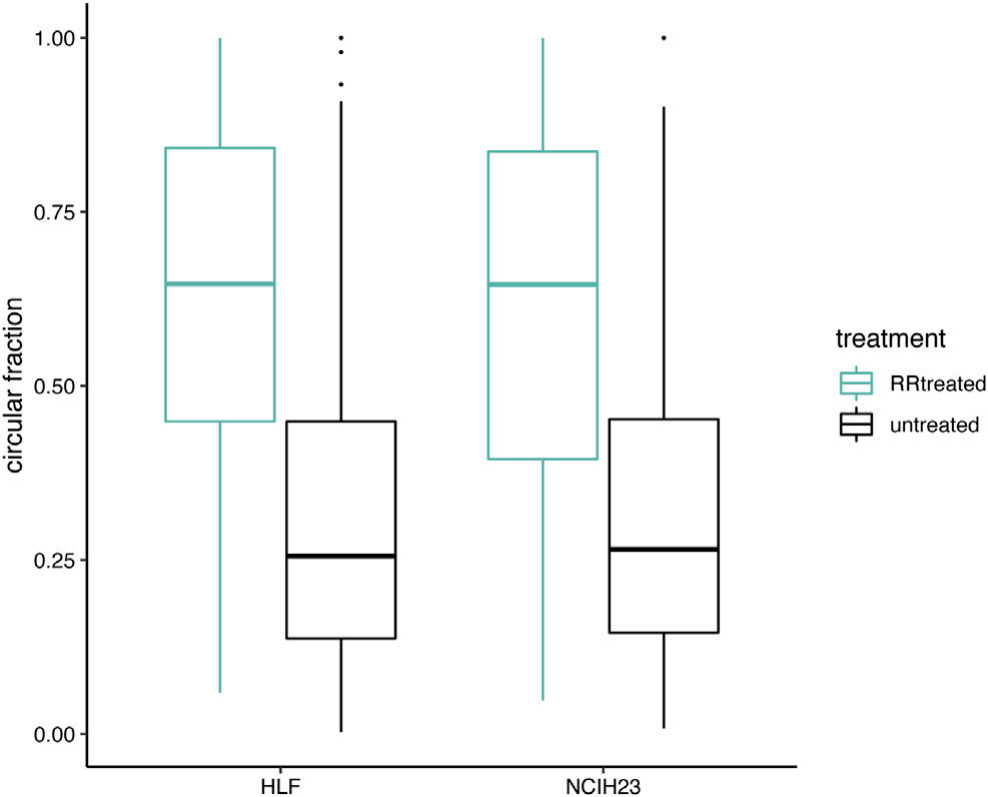
Circular transcript fractions increase after RNase R treatment. Circular fractions at back-splice level, based on flanking linear only junctions, are shown for back-splice junctions that were picked up (≥6 reads) in both conditions within one cell line (406 unique back-splice junctions in total; 305 in HLF and 223 in NCIH23). Pairwise comparisons within a cell line show a significant difference before (black) and after (green) RNase R treatment (Wilcoxon signed-rank test; *p* < 0.001).

RNase R digests linear RNAs while keeping circular RNA molecules intact. In Figure 4, we verified that the circRNA fractions, as computed by CiLiQuant, increased after RNase R treatment as expected. We observed a 2.5-fold and a 2.4-fold increase in circRNA fraction (back-splice level) in HLF and NCIH23 samples respectively when applying RNase R treatment. Similar enrichment was observed at gene level and this trend was also present at lower sequencing depths (data not shown). Of note, both at the gene and back-splice level, the circular fraction, does not always reach 100% after RNase R treatment. Possible explanations are that not all linear RNA is degraded by the RNase R treatment, or that circRNAs that span other forward-splice junctions may be missed because of low coverage, leading to misclassification of a splice junction as linear only.

## Discussion

Several RNA sequencing library preparation methods can pick up both linear and circular RNA transcripts. However, contrary to the clear circular RNA origin of back-splice reads, the linear or circular origin of forward-splice junctions is not always obvious. CiLiQuant classifies forward-splice junction reads as linear only or ambiguous depending on the overlap with detected circRNA transcripts. By correcting the sum of junction reads for the number of unique junctions, the linear only and back-splice junction reads can be directly compared. The entire pipeline is freely available on GitHub under MIT license (https://github.com/OncoRNALab/CiLiQuant), platform independent and can be initiated using a single command. Other count-based strategies that determine circular-to-linear RNA ratios rely on the maximally expressed transcript or on splice reads flanking the circRNA (Rybak-Wolf et al., 2015; Jakobi et al., 2019; Ma et al., 2019). However, these calculations can still be based on reads coming from both circular as well as linear transcripts in case other circRNAs span this region as well as shown in Figure 2. Moreover, the flexible input format of CiLiQuant also allows the usage of junction count files from various combinations of mappers and circRNA quantification tools. This enables users to look at circRNA transcript fractions using their preferred mapping and circRNA detection strategy. An alternative would be to use a model-based strategy that simultaneously quantifies linear and circular reads using a predefined pseudolinearized reference for circRNAs (Li et al., 2017).

The dual approach of CiLiQuant allows users to look at the circular versus linear RNA abundance from different perspectives. The confidence interval further provides the user with a level of uncertainty on the calculated fraction; e.g., a ratio of 2/2 has higher uncertainty than a ratio of 20/20 reads. This allows to study the effect of perturbations on alterations of this circular fraction, hinting at a causal relationship between the perturbans and splicing. As the output table includes the original counts, the user can still impose a minimum count threshold and look at absolute count differences. Possible applications include, but are not limited to, comparing different tissues, cell types or biomaterials with respect to the circRNA fraction of particular genes, or overall; identifying genes or regions with abundant circRNA enrichment; discovering interesting circRNAs for biomarker purposes. Finally, the ambiguous read category can help to determine the relative level of mixed (linear and circular RNA) signal in aggregated gene counts. Any gene with partially overlapping circRNAs or multi-exon circRNAs potentially suffers from ambiguous reads. These reads are often mistakenly considered as read evidence for linear transcripts (example in Figure 2). In case of sufficient sequencing depth, the linear only classification of CiLiQuant can be used as a starting point for differential expression analysis of linear RNA junction counts only. This approach avoids interference of circRNA derived reads in linear transcript differential expression analyses and it is similar to the current circRNA differential expression analyses that are based on back-splice junction counts only. A limitation of the CiLiQuant algorithm is that it depends on the detection of all circRNAs present in the sample to classify junction reads. In case a circRNA is not detected because of insufficient sequencing depth, the junction reads that fall within this circRNA may be misclassified as linear only. However, the obtained fractions could still be useful for relative comparisons of samples sequenced at the same depth.

The pipeline took 2 hours to process junction files generated from 10 M paired-end reads (single node run, maximum memory used: 370 M), this increased to 3 hours for 50 M paired-end read data (single node run, maximum memory used: 430 M). Parallelization is possible by splitting up the reference file, for example by chromosome.

In conclusion, the CiLiQuant pipeline distinguishes linear only from potentially mixed junction reads and determines the circular contribution in RNA sequencing data in a systematic and uniform way.

## Data Availability

The original contributions presented in the study are publicly available. This data can be found here: https://www.ncbi.nlm.nih.gov/bioproject/PRJNA789110.
